# Postoperative electrocardiography changes: To worry or not to worry

**DOI:** 10.1111/anec.13092

**Published:** 2023-11-20

**Authors:** Chihjen Lee, Janet Shin, Arash Bereliani, Liza Capiendo, Eiman Firoozmand, Roya Yumul

**Affiliations:** ^1^ Department of Anesthesiology Cedars‐Sinai Medical Center Los Angeles CA USA; ^2^ Department of Cardiology Cedars‐Sinai Medical Center Los Angeles CA USA; ^3^ Department of Surgery Cedars‐Sinai Medical Center Los Angeles CA USA

**Keywords:** biphasic T waves, breast implants, fluorouracil, point‐of‐care ultrasound, poor R‐wave progression, precordial lead placements, premature atrial contractions, Takotsubo cardiomyopathy, Trastuzumab, Wellens syndrome

## Abstract

Abnormal postoperative electrocardiograms are not uncommon, oftentimes leading to further cardiac workup especially when the findings are new and not easily explainable. A forty‐year‐old woman, with a history of left breast cancer status post bilateral mastectomies and reconstructions, presented for robot‐assisted low‐anterior resection secondary to rectal cancer. Postoperative electrocardiogram showed poor R wave progression, biphasic T waves in V2‐4, and possible anterior wall ischemia. Her electrocardiogram from 6 years ago was normal. No recent electrocardiogram was available for comparison. Initially, the abnormal postoperative electrocardiogram appeared worrisome. However, the patient was completely asymptomatic, and all vital signs were normal. Cardiac point‐of‐care ultrasound showed normal parasternal long and short axis views. The biphasic T waves in V2‐4 were suggestive of Wellens syndrome, but the accompanying poor R wave progression was not consistent with the diagnostic criteria. The anesthesiologist then remembered the patient's history of the presence of a left breast implant and suspected it might have caused the changes on the electrocardiogram. A literature search did find one publication that shows approximately 45% of patients with breast implants present with electrocardiogram changes, including poor R wave progression and negative T waves. Therefore, no further cardiac workup was ordered for our patient. She was discharged home 3 days later. Breast implants and electrocardiogram changes are a lesser‐known topic. Obtaining a pre‐operative electrocardiogram should be considered in patients with previous breast implants, to serve as a baseline for comparison if the patient were to need another electrocardiogram in the future.

## Graphical abstract

Approximately 45% of patients with the breast implants present with electrocardiogram changes, including poor R wave progression, negative T waves (V1‐4), etc.

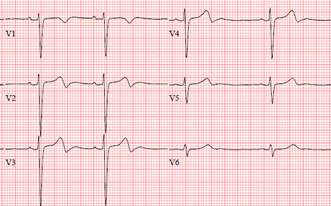

## 1 CASE DESCRIPTION

A 40‐year‐old woman presented to the hospital for a scheduled robotic low‐anterior resection with loop ileostomy, bilateral salpino‐oopherectomy, and flexible sigmoidoscopy for rectal adenocarcinoma discovered on colonoscopy about a year ago. Of note, the patient had a past medical history significant for triple positive infiltrating ductal carcinoma of the breast for which she had a bilateral mastectomy with bilateral breast reconstruction with tissue expanders about 5 years prior to presentation. A couple of months later, she had subsequent removal of the tissue expanders and placement of silicone breast implants. In addition to surgery, the patient received chemotherapy and HER2‐targeted treatment for breast cancer with docetaxel, carboplatin, trastuzumab, and pertuzumab. To monitor potential treatment‐related cardiotoxicity, the patient had received routine trans‐thoracic echocardiography (TTE) exams, including a stress echocardiogram, the results of which showed normal heart function and no evidence of ischemia. About 2 years prior to the presentation the patient had an episode of “chest wall throbbing” for which another TTE was performed. The results again showed normal cardiac function and no evidence of ischemia. The left ventricular ejection fraction was 64% with no regional wall motion abnormalities. The right ventricular systolic function was normal and there was no significant valvular heart disease. The chest wall throbbing had resolved completely by the time the patient presented to the hospital for surgery. Three months prior to the current surgery, the patient had completed total neoadjuvant therapy (capecitabine, radiation, and FOLFOX) for her locally advanced T3N1 rectal cancer. Our patient had no other known cardiac risk factors, such as diabetes mellitus, hypertension, chronic kidney disease, hyperlipidemia, sleep apnea, obesity, or smoking history. Her previous TTE exams were normal. She tolerated chemotherapy well, had great exercise tolerance, and denied any cardiac symptoms. The medical team cleared her for surgery.

**FIGURE 1:**
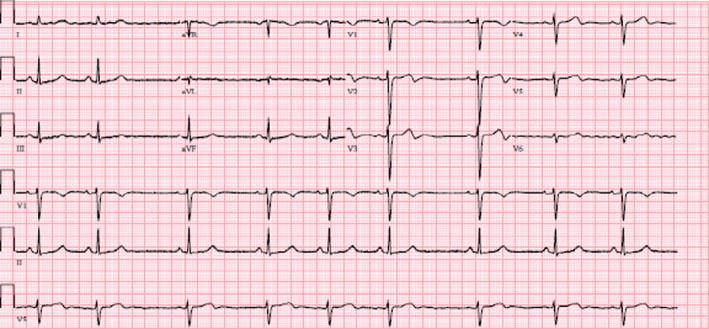
Postoperative EKG shows poor R wave progression and biphasic T waves in V2‐4. Also noted are occasional premature atrial contractions.

**FIGURE 2:**
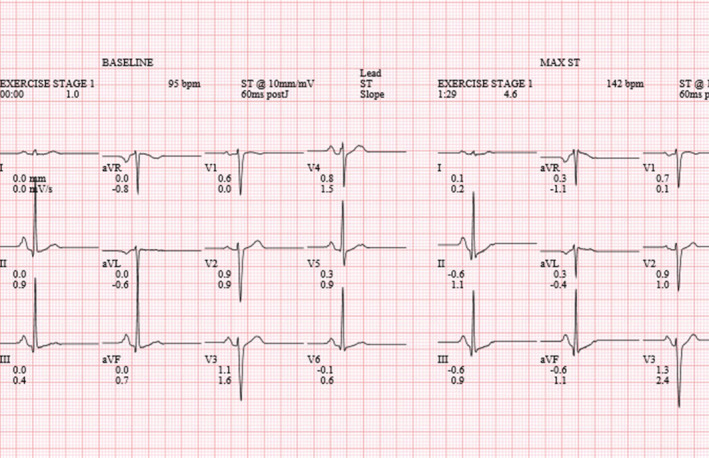
Preoperative EKG from stress echocardiogram prior to breast implant.

The patient underwent an uneventful intraoperative course with stable hemodynamics throughout the surgery. However, when the patient was in the post‐anesthesia‐care unit (PACU), the anesthesiologist was called regarding a sinus arrhythmia noted on telemetry. Upon bedside assessment, the patient had stable vital signs and denied any cardiac symptoms. A point of care cardiac ultrasound exam was performed in the parasternal short and long axis views which showed good right and left‐sided function with no regional wall motion abnormalities in those views. A 12‐lead electrocardiogram (EKG) was obtained. See Figure 1. For comparison, an old EKG from 6 years ago is shown in Figure 2.

The current EKG demonstrated several abnormalities suggesting ischemia, including poor R‐wave progression and biphasic T‐waves in the precordial leads. Given the possibility of incorrect lead placement, the EKG was repeated, with the same results. Most of the differential diagnoses for poor R‐wave progression (Anttila et al., 2010) such as anterior myocardial infarction, cardiomyopathy, conduction abnormalities, chronic lung disease, and anatomical abnormalities could be eliminated based on the patient's presentation, medical history, EKG, and bedside cardiac ultrasound exam. Wellens syndrome, as suggested by the biphasic T waves in precordial leads, was also ruled out later by its diagnostic criteria (Rhinehardt et al., 2002). The patient continued to be without any cardiac symptoms during her hospitalization. Postoperative basic metabolic panel was within normal limits and complete blood count showed slight anemia with hemoglobin of 10.7 g/dL and hematocrit of 33.3%, decreased from 12.4 g/dL and 37.3% prior to surgery. Due to the absence of symptoms, unremarkable lab work, and negative cardiac point‐of‐care ultrasound exam, no additional cardiac workup or blood test, e.g., troponin, was obtained. The patient was subsequently discharged in stable condition. Upon her postoperative follow‐up visit, the patient continued to remain physically active and denied any cardiac complaints.

## 2 DISCUSSION

With most of the differential diagnoses ruled out, what could have been causing the EKG findings in our patient? Our patient was a 40‐year‐old woman who had just undergone an uneventful robot‐assisted low‐anterior resection and resting comfortably in the PACU. The telemetry showed occasional atrial arrythmias that was later shown to be premature atrial contractions (PACs). PACs are mostly benign (discussed below) and do not warrant further investigation (Durmaz et al., 2020; Heaton & Yandrapalli, 2023). However, an EKG was ordered which showed poor R wave progression, biphasic T waves in V2‐4, and possible anterior wall ischemia. See Figure 1. Despite our patient being asymptomatic with a negative cardiac POCUS examination, the EKG findings warrant an explanation.

Our patient has a history of breast cancer surgery and bilateral breast implants. Could the breast implants have caused the EKG changes? A literature search confirmed the suspicion.

### 2.1 Breast implants and EKG changes

A study by Bun et al., 2019, investigated the possibility of electrocardiographic modifications induced by breast implants. In the study, EKGs were collected from 28 women with breast implants without any acute medical conditions and the EKGs were analyzed by two electrophysiologists. In total, 42% of the EKGs were considered abnormal by one electrophysiologist and 46% were considered abnormal by the other. The modifications were observed predominantly in the precordial leads as opposed to the limb leads. The abnormalities most frequently observed included poor R wave progression and negative T waves from V1 to V4, findings which were also present in our patient. With this information, the presence of the breast implant was a reasonable explanation for our patient's EKG abnormalities. Other findings noted in the study but not in our patient were ST depressions from V3 to V6, early repolarization in inferior leads, long QT (QTc = 480 or 500 ms), and left ventricular hypertrophy (Bun & Taghji, 2019).

The study proposed two mechanisms for the EKG modifications due to breast implants. One was lead misplacement, and the other was from the breast implant itself. It was hypothesized that the electrical vectors emanating from the heart could be deviated before reaching the skin by meeting the unexcitable silicone from the implant (Bun & Taghji, 2019). Although future studies are needed, a similar phenomenon is said to be present with ultrasound propagation in the presence of breast implants during echocardiography (Bun & Taghji, 2019). Currently, there is not sufficient literature to assess what the ideal EKG electrode placements would be for women with breast implants. However, the correct placement of precordial leads—under the breast instead of on the breast, should always be attempted in patients receiving electrocardiograms.

### 2.2 Poor R wave progression

The 12‐lead EKG consists of two sets of leads—limb leads and precordial leads. There are six precordial chest leads, named V1 through V6. The six leads are placed over the chest reflecting electrical activity over different regions of the heart. A wave of depolarization that heads toward an electrode will result in a positive deflection on the EKG.

Normally, the height of the R wave becomes progressively taller from V1 to V6. V1 to V3 should have an R wave of low amplitude and an S wave that is larger. V3 to V4 is the transition point to an R wave that has a larger amplitude than the S wave. Poor R wave progression (PRWP) denotes the absence of an increase in the amplitude of the R wave through the precordial leads. There are many causes of PRWP including lead misplacement, anterior wall myocardial infarction, left ventricular hypertrophy, Wolff–Parkinson–White syndrome, left bundle branch block, and chronic lung disease (Anttila et al., 2010).

### 2.3 Wellens syndrome

The presence of biphasic T waves can represent myocardial infarction and hypokalemia, differentiated by the former having an initial upward then downward deflection, and the latter having an initial downward deflection then an upward deflection. An interesting syndrome related to biphasic T waves is Wellens syndrome. This is a pattern of inverted or biphasic T waves in V2–V3 in the setting of ischemia‐related chest pain that is highly specific for critical stenosis of the left anterior descending artery (Rhinehardt et al., 2002). There are two patterns of T wave abnormalities present in Wellens syndrome: Type A has biphasic T waves with an initial positive deflection followed by a negative deflection and Type B has deeply and symmetrically inverted T waves. The T waves usually evolve over time from a Type A to a Type B pattern. It is interesting to note that Wellens syndrome could be ruled out in our patient, first from lack of any ischemia‐related chest pain, and from the fact that in true Wellens syndrome, inverted T waves are present in the setting of a normal R wave progression.

Wellens syndrome is characterized by EKG findings of biphasic or inverted T waves in V2–3 or V1–6. It is often associated with critical stenosis of the left anterior descending artery. Our patient's EKG also shows biphasic T waves in V2–4, resembling the Wellens syndrome. However, one of the diagnostic criteria for Wellens syndrome is preserved R wave progression (Rhinehardt et al., 2002), unlike the poor R wave progression noted in our patient. Therefore, our patient's EKG is not consistent with that of Wellens syndrome. In other words, in Wellens syndrome, the R waves and T waves in precordial leads are discordant. Since the R waves do not progress in our EKG, it is conceivable for the T waves “not to progress” as well, and to become biphasic.

### 2.4 Premature atrial contractions

PACs are caused by early depolarization of atrial tissue. PACs can often be present in patients with or without known structural heart disease and, unless they cause significant symptoms, do not require treatment. PACs therefore are generally considered safe (Durmaz et al., 2020; Heaton & Yandrapalli, 2023). In some studies, however, they have been shown to be associated with future occurrences of atrial fibrillation, especially when the foci of the PACs are from the pulmonary veins. In a study by Durmaz et al. 2019, they found that frequent PACs are strongly and independently associated with the future development of atrial fibrillation and that this association was higher in patients with frequent PACs defined as more than 3000 PACs in a 24‐h period (Durmaz et al., 2020).

### 2.5 Precordial lead placements

The correct placement of the precordial leads is important to prevent falsely abnormal EKG patterns. The correct placement is as follows:

V1 lead should be placed in the fourth intercostal space, to the right of the sternum.
V2 lead should be placed in the fourth intercostal space, to the left of the sternum.
V3 lead should be placed diagonally between V2 and V4.
V4 lead should be placed between ribs 5 and 6 in the midclavicular line.
V5 lead should be placed between ribs 5 and 6 in the anterior axillary line.
V6 lead should be placed between ribs 5 and 6 in the midaxillary line.

A study by Rehman and Rehman, 2020, demonstrated that precordial EKG lead mispositioning can lead to significantly abnormal EKG patterns that lead to unnecessary further cardiovascular workup, including even a coronary angiography (Rehman & Rehman, 2020). This leads to increased costs and risks for the patient in addition to a significant financial burden on the economy (Rehman & Rehman, 2020). The study mentioned the common inaccurate placement of chest leads, especially leads V1 and V2, and that the most common chest lead misplacement error leads to poor R‐wave progression or reversed R‐wave progression, often interpreted as an acute myocardial infarction.

### 2.6 Chemotherapy and cardiac function

In total, 15% of breast cancers are HER2 positive. These patients may receive chemotherapy and HER2‐targeted agents, both potentially causing cardiotoxicity. Chemotherapy, e.g., anthracycline, may cause irreversible cardiac damage, which presents acutely as EKG abnormalities within a week or chronically as cardiac dysfunction within 1 year or later after completion of therapy (Doyle et al., 2005; Jerusalem et al., 2019). HER2‐targeted agents, e.g., Trastuzumab, cause reversible cardiac damage resulting in an asymptomatic decrease in EF (Jerusalem et al., 2019). The incidences are as follows (Jerusalem et al., 2019):

Trastuzumab: 5%
Trastuzumab + paclitaxel: 13%
Trastuzumab + anthracycline + cyclophosphamide: 27%

Our patient was HER2 positive, and she received docetaxel, carboplatin, trastuzumab, and pertuzumab. Due to the history of receiving these potentially cardiotoxic drugs, her cardiac function had been followed by echocardiography despite not having a history of cardiac risk factors. All of our patient's previous TTEs were normal.

Our patient also received chemotherapy and radiation (total neoadjuvant therapy) for her T3N1 locally advanced rectal cancer prior to surgery. The regimen included capecitabine (Xeloda) and eight cycles of FOLFOX (folinic acid, fluorouracil, and oxaliplatin). Capecitabine is an oral prodrug of 5‐fluorouracil and has been incorporated into multiple cancer treatments due to its ease of administration as well as efficacy comparable to 5‐fluorouracil. Next to anthracyclines, 5‐fluorouracil is the second most common chemotherapy agent causing cardiotoxicity. The cardiotoxicity from 5‐fluorouracil has been thought to stem from coronary vasospasm resulting in angina and even myocardial infarction; however, some literature also describes a cardiomyopathic picture as well. The incidence of 5‐fluorouracil‐induced cardiotoxicity ranges in the literature from 1% to 19%. The cardiotoxicity from 5‐fluorouracil is often transient and reversible after discontinuation of the drug (Shiga & Hiraide, 2020). Our patient had no known cardiac risk factors and had previous normal TTE exams. She tolerated chemotherapy well, had great exercise tolerance and denied any cardiac symptoms. She was cleared for surgery by the medical team.

### 2.7 Chemotherapy and EKG changes

A study by Liang, et al., studied electrocardiographic changes in patients who had chemotherapy in patients with breast cancer (Liang et al., 2020). They found that the incidence of abnormal EKG increased from 43.8% to 65.6% indicating abnormalities in depolarization and repolarization. The major abnormalities were due to a higher proportion of patients having fragmented QRS, which is a surrogate marker of myocardial conduction delay possibly from myocardial fibrosis (Liang et al., 2020). Other abnormalities included those related to heart rate, P wave dispersion, QTc, and RR interval. There were no significant changes in R wave progression or T wave inversion noted in this study. In addition, our patient did not show any echocardiographic evidence of cardiac toxicity from her breast cancer treatment, making it less likely that her EKG findings were due to her breast cancer chemotherapy.

Another differential diagnosis is Takotsubo cardiomyopathy due to chemotherapy. The typical clinical presentation includes chest pain, dyspnea, and occasionally hemodynamic compromise. The EKG shows ST elevations, T wave inversions, and occasionally pathologic Q waves. Troponin levels are often mildly elevated. Echocardiography typically shows apical hypokinesis and ballooning of the left ventricle during systole. Sundaravel et al. described a patient with FOLFOX‐induced Takotsubo cardiomyopathy treated with Impella assist device (Sundaravel et al., 2017). Takotsubo cardiomyopathy was ruled out in our patient by clinical presentations, normal preoperative chest X‐ray, and negative findings of postoperative point of care cardiac ultrasound.

### 2.8 Indications of preoperative EKGs

For which patients is a preoperative EKG indicated? According to the 2014 ACC/AHA Guidelines on Perioperative Cardiovascular Evaluation and Management of Patients Undergoing Noncardiac Surgery, functional status is a reliable predictor of perioperative and long‐term cardiac events, and, in highly functional asymptomatic patients, preoperative cardiac testing can be foregone prior to surgery (Fleisher et al., 2014). According to these guidelines, the value of the preoperative EKG increases with the risk of the surgery, especially in patients with known cardiac conditions such as coronary artery disease, arrhythmias, peripheral arterial disease, cerebrovascular disease, or other significant structural heart disease. For asymptomatic patients without risk factors such as diabetes mellitus, hypertension, chronic kidney disease, hyperlipidemia, sleep apnea, or obesity, a preoperative 12‐lead EKG may be used as a baseline for comparison. Our patient did not have significant risk factors or known cardiac conditions. Although she had completed chemotherapy with potential cardiotoxic adverse effects, she had received routine echocardiographic workups during her treatments, which consistently demonstrated normal cardiac function. This was also clinically correlated with the patient's excellent functional status. Our patient has no above‐listed risk factors and her BMI was 22.67 kg/m^2^.

### 2.9 Economy of excessive cardiac workup

As stated earlier, falsely abnormal EKG patterns do not come without consequences. Patients may be subjected to unnecessary further workup in the form of echocardiograms, cardiac stress tests, and lab work. Many of these tests have the potential to lead to false positive results, leading to more invasive workups such as coronary angiography. As the study by Rehman and Rehman, 2020, shows, these mistakes can be costly (Rehman & Rehman, 2020). Per their estimates, EKG lead mispositioning could be wasting roughly $3.2 billion in the United States annually (Rehman & Rehman, 2020).

## AUTHOR CONTRIBUTIONS

Chihjen Lee: conceptualization, manuscript preparation, review, and editing. Janet Shin: manuscript preparation, review, and editing, visualization. Arash Bereliani: manuscript review and editing, final approval of the version to be published. Liza Capiendo: provision of study patient, manuscript review and editing. Eiman Firoozmand: provision of study patient, manuscript review and editing. Roya Yumul: conceptualization, project administration, supervision.

## CONFLICT OF INTEREST STATEMENT

No conflict of interest.

## ETHICS STATEMENT

Not applicable.

## PATIENT CONSENT STATEMENT

A written consent for publication was obtained from the patient.

## Data Availability

Data sharing not applicable to this article as no datasets were generated or analysed during the current study.
